# Cynaroside Attenuates Ethinylestradiol-Induced Cholestatic Liver Injury by Modulating the FXR/BSEP/CYP7A1/SHP Axis

**DOI:** 10.3390/ijms27198839

**Published:** 2026-10-03

**Authors:** Amany M. Hamed, Doha M. Al-Nouri, Nadia S. Mahrous, Asmaa Sabry Bassit, Azza M. A. Abouelella, Asmaa Hassan, Gehad S. Mokhtar, Ghada M. Ahmad, Ahmed S. Osman, Ahmed R. H. Ahmed, Aya A. E. Mahgoob, Shymaa A. Thabet, Hany M. R. Abdel-Latif, Ghalia Shamlan, Haifeng Zhao, Ammar Al-Farga, Safaa S. Soliman

**Affiliations:** 1 Chemistry Department, Faculty of Science, Sohag University, Sohag 82524, Egypt; 2 Department of Food and Nutrition Sciences, College of Food and Agriculture Sciences, King Saud University, Riyadh 11495, Saudi Arabia; 3 Department of Zoology, Faculty of Science, South Valley University, Qena 83523, Egypt; 4 Anatomy & Embryology Department, Faculty of Medicine, Sohag University, Sohag 82524, Egypt; 5 Department of Clinical Pharmacology, Faculty of Medicine, Sohag University, Sohag 82524, Egypt; 6 Department of Physiology, Faculty of Medicine, Sohag University, Sohag 82524, Egypt; 7 Department of Molecular Biology, Faculty of Applied Biotechnology, Merit University, Sohag 82755, Egypt; 8 Department of Medical Laboratory Technology, Faculty of Applied Health Sciences Technology, Pharos University in Alexandria, Canal El Mahmoudia Street, Beside Green Plaza Complex, Alexandria 21648, Egypt; ghada.mohammad@pua.edu.eg; 9 Department of Biochemistry, Faculty of Veterinary Medicine, Sohag University, Sohag 82524, Egypt; 10 Department of Pathology, Faculty of Medicine, Sohag University, Sohag 82524, Egypt; 11 Technology of Medical Laboratory Department, Higher Technology Institute of Applied Health Sciences, Badr Academy for Technology and Sciences, Badr City 11829, Egypt; 12 Central Research Center, Faculty of Medicine, Sohag University, Sohag 82524, Egypt; 13 Department of Poultry and Fish Diseases, Faculty of Veterinary Medicine, Alexandria University, Alexandria 26571, Egypt; hmhany@alexu.edu.eg; 14 School of Food Science and Engineering, South China University of Technology, Guangzhou 510640, China; 15 Department of Biological Sciences, College of Science, University of Jeddah, Jeddah 21589, Saudi Arabia; 16 Department of Zoology, Faculty of Science, Minia University, Minia 61519, Egypt

**Keywords:** cholestasis, ethinylestradiol, cynaroside, hepatoprotection, NF-κB, Nrf2, FXR

## Abstract

Cholestatic liver diseases are characterized by disrupted bile acid homeostasis, oxidative stress, and inflammation, ultimately leading to progressive hepatic injury. Although ursodeoxycholic acid (UDCA) remains the standard pharmacological therapy, its therapeutic efficacy is limited in several cholestatic conditions. This study investigated the hepatoprotective effects of cynaroside (CYN), a naturally occurring flavonoid glycoside, against ethinylestradiol (EE)-induced cholestatic liver injury and explored its underlying molecular mechanisms. Female Wistar rats were randomly allocated into four groups (*n* = 7): control, EE, EE + CYN, and EE + UDCA. Liver function indices, bile acids, cholesterol, oxidative stress and inflammatory biomarkers, histopathology, immunohistochemistry, Western blotting, qRT-PCR, and molecular docking analyses were performed. EE administration induced pronounced cholestatic liver injury, as evidenced by impaired liver function, hepatic accumulation of bile acids and cholesterol, oxidative imbalance, inflammatory responses, and marked histopathological alterations. CYN significantly ameliorated these biochemical and histological abnormalities, reduced hepatic bile acid and cholesterol accumulation, restored redox homeostasis, and attenuated inflammatory responses. Mechanistically, CYN modulated the hepatic FXR/BSEP/CYP7A1/SHP regulatory network, modulated hepatic FXR and Nrf2 protein expression, suppressed NF-κB activation, and favorably regulated TNF-α, IL-1β, and BCL2 expression. Histopathological and immunohistochemical findings further confirmed substantial preservation of hepatic architecture following CYN treatment. Molecular docking revealed favorable interaction of CYN with the FXR ligand-binding domain, with a predicted binding free energy of −8.18 kcal/mol, comparable to that of UDCA (−7.95 kcal/mol). Cynaroside attenuated EE-induced cholestatic liver injury by improving bile acid homeostasis and mitigating oxidative and inflammatory responses, potentially by modulating FXR-associated signaling and its downstream regulatory network. Collectively, the results provide a rationale for continued investigation of cynaroside in the context of cholestatic liver injury.

## 1. Introduction

Cholestatic liver diseases encompass a diverse spectrum of hepatic disorders in which the synthesis, secretion, or transport of bile is disrupted, resulting in decreased bile flow and retention of bile components in the liver [1]. The subsequent accumulation of hydrophobic bile acids exerts cytotoxic effects on hepatocytes and cholangiocytes, promoting progressive liver damage that may advance to chronic inflammation, fibrosis, cirrhosis, and hepatic failure if left untreated [2]. Owing to their multifactorial pathogenesis and the limited efficacy of currently available therapies, cholestatic disorders continue to represent a significant clinical burden. Recent studies have identified disturbed bile acid homeostasis as a key pathogenic mechanism rather than a secondary manifestation of cholestasis. Excessive intrahepatic bile acids trigger oxidative stress, mitochondrial impairment, inflammatory signaling, and programmed cell death, thereby accelerating disease progression [3,4]. Accordingly, therapeutic strategies aimed at re-establishing bile acid homeostasis have become a viable strategy for limiting liver injury and improving clinical outcomes in cholestatic liver diseases [5].

Nuclear receptors must work in unison to maintain bile acid balance, membrane transporters, and enzymes involved in bile acid synthesis and disposition. Among these regulators, the farnesoid X receptor (FXR) plays a central role in sensing intracellular bile acid levels and orchestrating adaptive responses that preserve hepatic bile acid balance [6]. By simultaneously decreasing their production and increasing their biliary excretion, modulation of FXR-related signaling starts a transcriptional pathway that restricts the buildup of bile acids. The bile salt export pump (BSEP), an ATP-dependent canalicular transporter that facilitates the secretion of bile acids from hepatocytes into bile, is a key player in this defense mechanism [7]. Upregulation of BSEP through FXR signaling promotes efficient bile acid elimination and minimizes hepatocellular toxicity. In parallel, FXR suppresses the expression of cholesterol 7α-hydroxylase (CYP7A1), the rate-limiting enzyme responsible for the classical pathway of bile acid biosynthesis, thereby preventing excessive bile acid production [8]. Impairment of the FXR–BSEP–CYP7A1 signaling network disrupts bile acid homeostasis, favors intrahepatic bile acid retention, and contributes to the initiation and progression of cholestatic liver injury. Consequently, pharmacological modulation of this regulatory axis has emerged as a promising therapeutic strategy for the management of cholestatic liver diseases [7,9,10].

Among the available experimental models of cholestatic liver injury, the 17α-ethinylestradiol (EE)-induced model has been widely employed because it reproduces several pathological and biochemical features of estrogen-related cholestasis, including hormone replacement therapy, oral contraceptive use, and pregnancy-related disorders [11]. Administration of EE disrupts hepatobiliary bile acid transport by impairing the expression and function of canalicular transport proteins, altering hepatocyte membrane properties, and triggering inflammatory responses that culminate in intrahepatic bile acid retention and liver injury [12]. Experimental evidence further indicates that EE interferes with FXR-mediated signaling, resulting in reduced expression of the bile salt export pump (BSEP) and disturbed regulation of cholesterol 7α-hydroxylase (CYP7A1), thereby aggravating bile acid imbalance [13]. The subsequent accumulation of cytotoxic bile acids stimulates inflammatory pathways, particularly NF-κB activation, and enhances the production of pro-inflammatory cytokines, creating a self-perpetuating cycle of inflammation and hepatocellular damage [14,15].

Ursodeoxycholic acid (UDCA) remains the established first-line pharmacological therapy for primary cholestatic liver diseases and continues to serve as an important therapeutic reference [16]. However, a substantial proportion of patients show an incomplete biochemical response or continue to develop progressive liver injury, highlighting the need for additional therapeutic strategies. Accordingly, several mechanism-based approaches have emerged, including FXR agonists, such as obeticholic acid, PPARα/δ agonists, such as elafibranor, and ileal bile acid transporter (IBAT/ASBT) inhibitors, which target bile acid synthesis, detoxification, transport, and enterohepatic circulation [17,18]. Despite their therapeutic potential, limitations related to adverse effects, variable clinical responses, and treatment-related considerations underscore the need for novel, better-tolerated agents that restore bile acid homeostasis while attenuating oxidative and inflammatory injury. In this context, modulation of the FXR pathway, a central regulator of hepatic bile acid synthesis and transport, represents an attractive therapeutic strategy for protecting against cholestatic liver injury [19,20,21].

Naturally derived bioactive compounds have attracted considerable interest as potential therapeutic candidates for liver diseases because of their broad spectrum of biological activities and relatively favorable safety profiles. Among these compounds, cynaroside (luteolin-7-O-glucoside), a flavone glycoside widely distributed in numerous medicinal plants, including Cynara scolymus, has been shown to have a variety of pharmacological actions, including hepatoprotective, lipid-modulating, anti-inflammatory, and antioxidant properties [22]. Previous investigations have demonstrated that cynaroside mitigates inflammatory responses by inhibiting NF-κB signaling, suppresses NLRP3 inflammasome activation, and protects hepatic tissues against oxidative damage. In addition, growing evidence indicates that this flavonoid participates in regulating cholesterol metabolism and bile acid synthesis, partly by modulating CYP7A1 expression, suggesting a potential role in preserving bile acid homeostasis and limiting cholestatic liver injury [23].

Luteolin, the aglycone of cynaroside, has also attracted considerable interest as a hepatoprotective flavonoid. According to experimental research, luteolin can reduce cholestatic liver damage by regulating inflammatory signals, oxidative stress, and bile acid-related pathways. Importantly, cynaroside is the 7-O-glucoside derivative of luteolin, and its biological effects may therefore be influenced by its metabolic conversion and/or intestinal deglycosylation to luteolin [24,25]. Nevertheless, the pharmacological activity of the glycosylated compound cannot necessarily be assumed to be identical to that of its aglycone, as glycosylation may influence intestinal absorption, metabolism, bioavailability, and tissue exposure. Direct evaluation of cynaroside in estrogen-induced cholestatic liver injury is therefore important for distinguishing the activity of the parent glycoside from that reported for luteolin [26,27].

Although both cynaroside and its aglycone luteolin have demonstrated hepatoprotective and anti-inflammatory activities in experimental models, their relative effects and mechanisms in estrogen-induced cholestatic liver injury remain insufficiently characterized. In particular, it remains unclear whether cynaroside can modulate bile acid homeostasis through coordinated regulation of the FXR/BSEP/CYP7A1/SHP network while simultaneously attenuating oxidative stress and inflammatory signaling in this specific model.

Therefore, the current work sought to identify the molecular processes behind cynaroside’s activity and examine its hepatoprotective effects in a rat model of cholestatic liver damage caused by 17α-ethinylestradiol. The effects of cynaroside on hepatic oxidative stress and inflammatory signaling, as well as the regulation of bile acid homeostasis via the FXR/BSEP/CYP7A1/SHP regulatory network, were given special attention. The above findings were also interpreted in light of the available data about luteolin-mediated protection against cholestatic liver injury, given the documented biological activities of luteolin, the aglycone of cynaroside.

## 2. Results

### 2.1. Effect of Cynaroside on Liver Weight and Liver-to-Body-Weight Ratio

**Figure 1:**
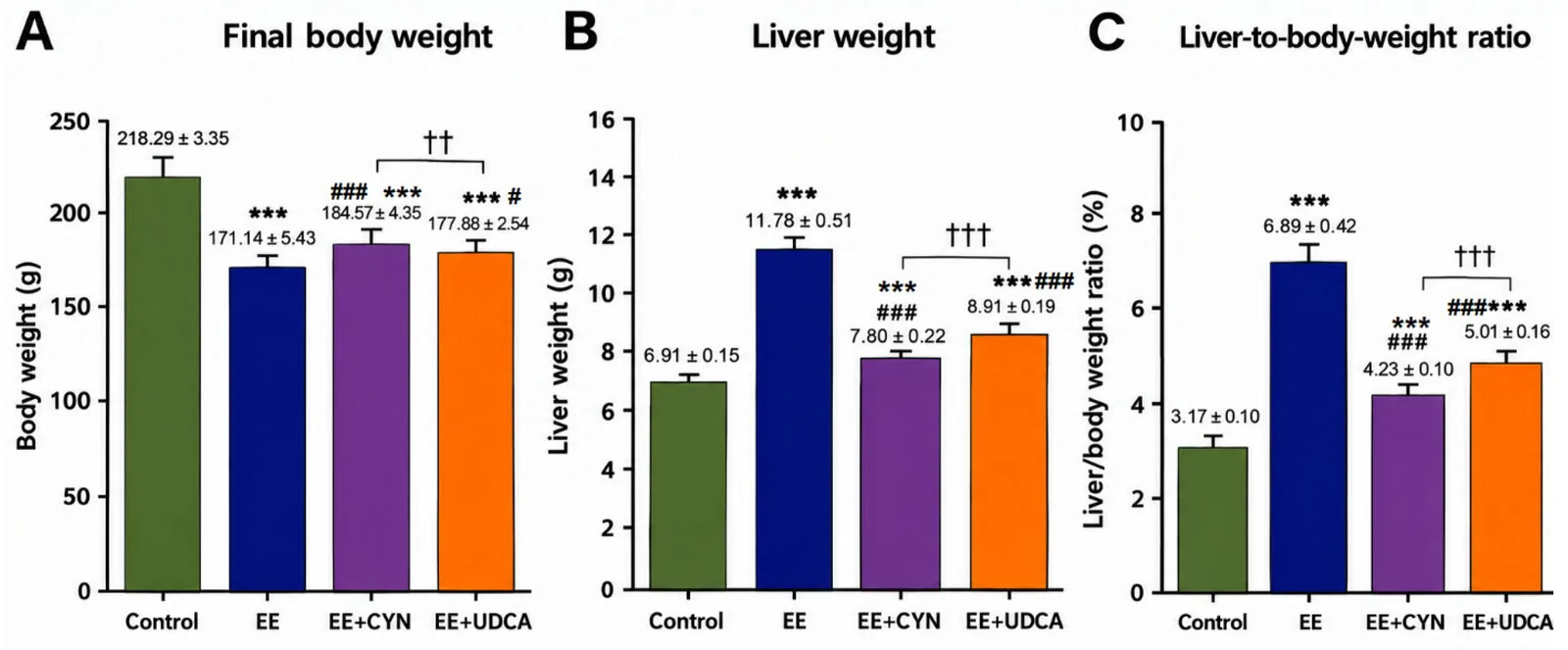
Effects of EE, CYN, and UDCA on final body weight, liver weight, and liver-to-body-weight ratio in female Wistar rats. (**A**) Final body weight before dissection, (**B**) absolute liver weight, and (**C**) liver-to-body-weight ratio. Data are presented as mean ± SD (*n* = 7 animals per group). We performed statistical analyses using one-way ANOVA followed by Tukey’s multiple-comparison test. *** *p* < 0.001 versus the control group; ^#^ *p* < 0.05 and ^###^ *p* < 0.001 versus the EE group; ^††^ *p* < 0.01 and ^†††^ *p* < 0.001 for the CYN versus UDCA comparison.

As shown in Figure 1, administration of 17α-ethinylestradiol (EE) significantly increased absolute liver weight compared with the control group (*** *p* < 0.001), accompanied by a significant reduction in final body weight (*** *p* < 0.001). Consequently, the liver-to-body-weight ratio was markedly increased in EE-treated animals compared with the control group (*** *p* < 0.001), consistent with pronounced hepatomegaly.

Treatment with cynaroside (CYN) significantly reduced liver weight compared with the EE group (^###^ *p* < 0.001) and significantly increased final body weight relative to EE-treated animals (^###^ *p* < 0.001). However, both absolute liver weight and the liver-to-body-weight ratio remained significantly higher than those of the control group (*** *p* < 0.001), indicating that CYN substantially attenuated, but did not completely normalize, the EE-associated increase in liver mass.

Similarly, ursodeoxycholic acid (UDCA) significantly reduced liver weight compared with the EE group (^###^ *p* < 0.001) and increased final body weight relative to EE-treated animals (^#^ *p* < 0.05). Nevertheless, both liver weight and the liver-to-body-weight ratio remained significantly higher than those of the control group (*** *p* < 0.001).

Overall, both CYN and UDCA significantly attenuated the increase in absolute and relative liver weight induced by EE. Direct comparison between the CYN- and UDCA-treated groups revealed significant differences in final body weight (*p* = 0.0056), absolute liver weight (*p* < 0.001), and liver-to-body-weight ratio (*p* < 0.001). CYN-treated animals showed a higher final body weight and lower absolute liver weight and liver-to-body-weight ratio than UDCA-treated animals. Nevertheless, neither treatment completely restored these parameters to control values.

### 2.2. Effect of Cynaroside on Liver Function Biomarkers

As shown in Table 1, EE administration induced pronounced alterations in serum liver function biomarkers compared with the control group. EE significantly increased AST, ALT, ALP, and GGT activities, as well as direct and total bilirubin levels (*p* < 0.001 for all parameters), while significantly reducing total protein and albumin concentrations (*p* < 0.001). These findings indicate substantial biochemical disturbances associated with EE-induced hepatic and cholestatic injury.

CYN treatment markedly attenuated the EE-induced alterations in liver function biomarkers. Compared with the EE group, CYN significantly reduced AST, ALT, ALP, and GGT activities (^###^ *p* < 0.001 for all parameters), as well as direct and total bilirubin levels (^###^ *p* < 0.001). Conversely, CYN significantly increased total protein concentration (^##^ *p* < 0.01) and albumin concentration (^###^ *p* < 0.001) relative to the EE group. Notably, albumin concentration in the CYN-treated group was not significantly different from that of the control group (ns), whereas the remaining assessed biomarkers remained significantly different from the control group, with significance levels ranging from *p* < 0.05 to *p* < 0.01. Thus, CYN substantially attenuated the EE-associated biochemical alterations, with restoration toward control values differing among individual parameters.

UDCA treatment similarly ameliorated the EE-induced alterations in serum liver function biomarkers. Compared with the EE group, UDCA significantly decreased AST, ALT, ALP, and GGT activities (^###^ *p* < 0.001), together with direct and total bilirubin levels (^##^ *p* < 0.01). UDCA also significantly increased total protein concentration compared with the EE group (^##^ *p* < 0.01). In contrast, albumin concentration did not differ significantly between the UDCA and EE groups (ns). Relative to the control group, AST, ALT, ALP, GGT, total protein, albumin, direct bilirubin, and total bilirubin remained significantly altered (*p* < 0.01–0.001). Overall, UDCA attenuated the biochemical disturbances induced by EE, although several parameters remained significantly different from control values.

Collectively, these findings demonstrate that both CYN and UDCA mitigated the EE-induced alterations in serum markers of hepatocellular injury, cholestasis, bilirubin metabolism, and protein status. The comparatively greater restoration of albumin toward control values in the CYN-treated group was accompanied by significant improvement in the other measured biomarkers, although most parameters did not completely return to control levels.

### 2.3. Effect of Cynaroside on Bile Acid and Cholesterol Levels

**Figure 2:**
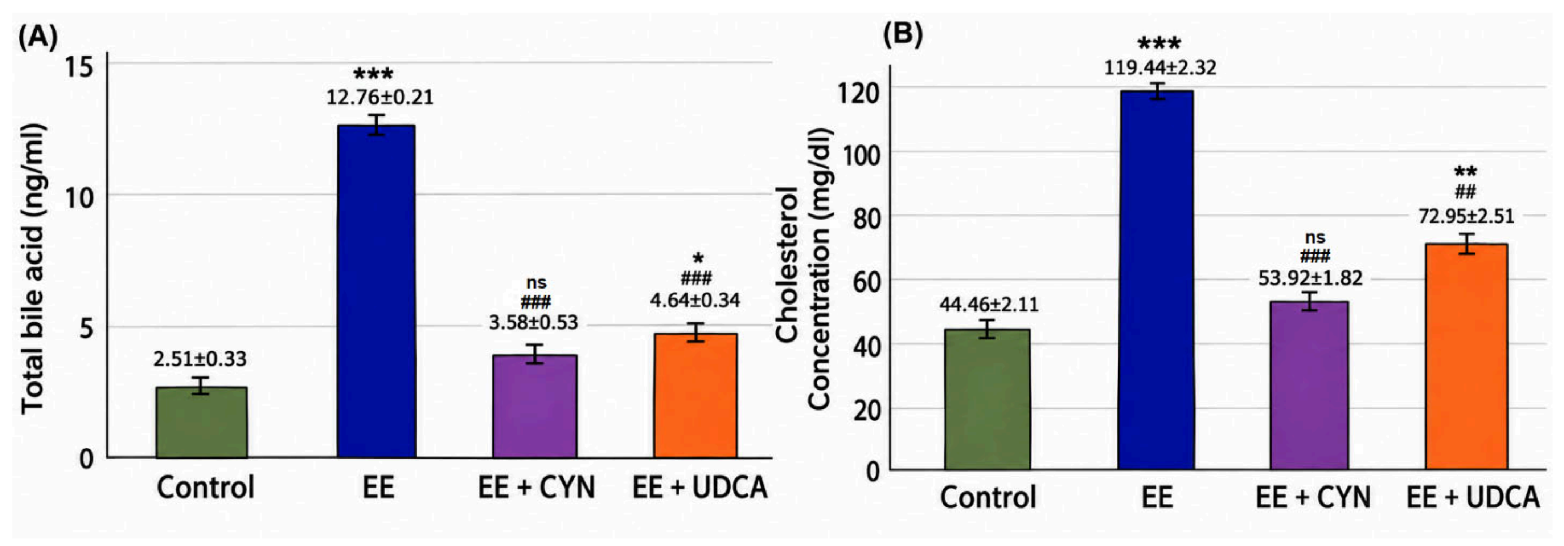
Effect of CYN and UDCA on bile acid and cholesterol levels in EE-induced cholestasis. (**A**) Total bile acid (TBA) levels and (**B**) serum cholesterol concentrations in control, ethinylestradiol (EE), EE treated with cynaroside (CYN), and EE treated with ursodeoxycholic acid (UDCA) groups. Data are presented as mean ± SD (*n* = 7). Statistical comparisons were performed using one-way ANOVA followed by Tukey’s post hoc test. * Significant vs. control group (* *p* < 0.05); ** *p* < 0.01; *** *p* < 0.001. ^#^ Significant vs. EE group; ^##^ *p* < 0.01; ^###^ *p* < 0.001. ns: non-significant vs. control group.

As shown in Figure 2, administration of 17α-ethinylestradiol (EE) resulted in significant increases in serum total bile acid (TBA) and cholesterol levels compared with the control group (*p* < 0.001), confirming the successful induction of cholestatic liver injury.

Treatment with cynaroside (CYN) significantly reduced both TBA and cholesterol levels relative to the EE group (^#^ *p* < 0.01 and ^#^ *p* < 0.001, respectively). Importantly, neither parameter differed significantly from the corresponding control values (ns), indicating restoration of bile acid and cholesterol homeostasis.

Similarly, ursodeoxycholic acid (UDCA) significantly decreased serum TBA and cholesterol levels compared with the EE group (^#^ *p* < 0.01). However, both parameters remained significantly higher than those observed in the control group (*p* < 0.05–0.01), indicating incomplete normalization under the present experimental conditions.

Overall, both CYN and UDCA attenuated EE-induced disturbances in bile acid and cholesterol metabolism, whereas only CYN restored these parameters to levels comparable with those of the control group.

### 2.4. Effect of Cynaroside on Oxidative Stress Markers

As presented in Table 2, EE administration significantly increased hepatic malondialdehyde (MDA) levels and decreased reduced glutathione (GSH) content compared with the control group (*p* < 0.001 for both parameters), indicating marked disruption of the hepatic redox balance following EE exposure.

CYN treatment significantly attenuated the EE-induced oxidative alterations. CYN significantly reduced hepatic MDA levels compared with the EE group (^###^ *p* < 0.001), with no significant difference from the control group (ns). In parallel, CYN significantly increased GSH content relative to the EE group (^###^ *p* < 0.001), although GSH remained significantly different from the control group (*p* < 0.05).

UDCA also significantly ameliorated the EE-induced oxidative disturbances. Compared with the EE group, UDCA significantly reduced MDA levels (^##^ *p* < 0.01) and increased GSH content (^#^ *p* < 0.05). However, both MDA and GSH levels remained significantly different from the control group (*p* < 0.01).

Overall, these findings demonstrate that both CYN and UDCA attenuated the oxidative stress associated with EE exposure, with CYN showing a greater restoration of MDA levels toward those observed in the control group.

### 2.5. Effect of Cynaroside on Inflammatory Markers

**Figure 3:**
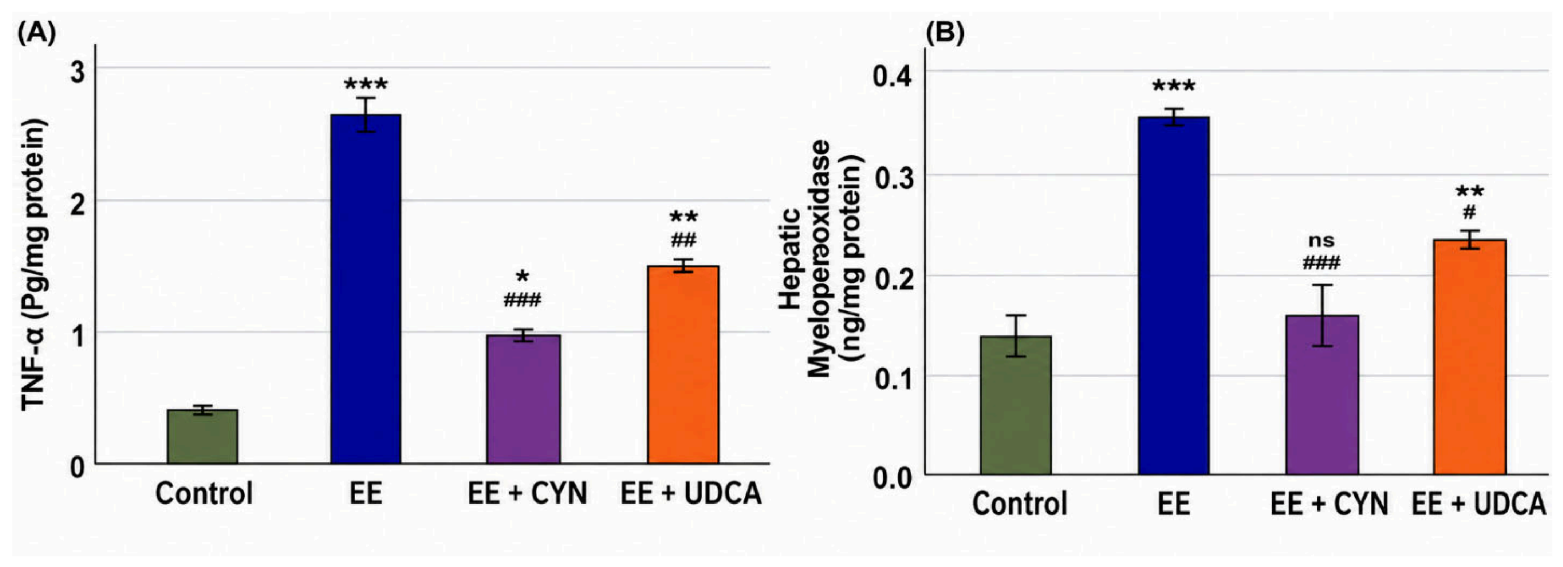
Effect of CYN and UDCA on hepatic inflammatory markers in EE-induced liver injury. (**A**) Serum tumor necrosis factor-alpha (TNF-α) and (**B**) hepatic myeloperoxidase (MPO) levels in control, ethinylestradiol (EE), EE treated with cynaroside (CYN), and EE treated with ursodeoxycholic acid (UDCA) groups. Data are expressed as mean ± SD (*n* = 7). Statistical comparisons were performed using one-way ANOVA followed by Tukey’s post hoc test. * Significant vs. control group (* *p* < 0.05); ** *p* < 0.01; *** *p* < 0.001. ^#^ Significant vs. EE group (^#^ *p* < 0.05); ^##^ *p* < 0.01; ^###^ *p* < 0.001. ns: non-significant vs. control group.

As shown in Figure 3, administration of 17α-ethinylestradiol (EE) markedly increased serum tumor necrosis factor-α (TNF-α) levels and hepatic myeloperoxidase (MPO) activity compared with the control group (*p* < 0.001), indicating a robust inflammatory response.

Treatment with cynaroside (CYN) significantly attenuated these inflammatory changes relative to the EE group (^#^ *p* < 0.001). Hepatic MPO activity was restored to a level that was not significantly different from that of the control group (ns), whereas serum TNF-α concentrations, despite being markedly reduced, remained slightly but significantly higher than the control values (*p* < 0.05).

Similarly, treatment with ursodeoxycholic acid (UDCA) significantly decreased both serum TNF-α levels and hepatic MPO activity compared with the EE group (^#^ *p* < 0.05–0.01). However, both inflammatory markers remained significantly elevated relative to the control group (*p* < 0.01), indicating incomplete resolution of the inflammatory response.

Overall, both CYN and UDCA markedly attenuated EE-induced inflammation, whereas only CYN restored hepatic MPO activity to a level comparable with that of the control group.

### 2.6. Effect of Cynaroside on Hepatic Nrf2 and FXR Protein Expression

**Figure 4:**
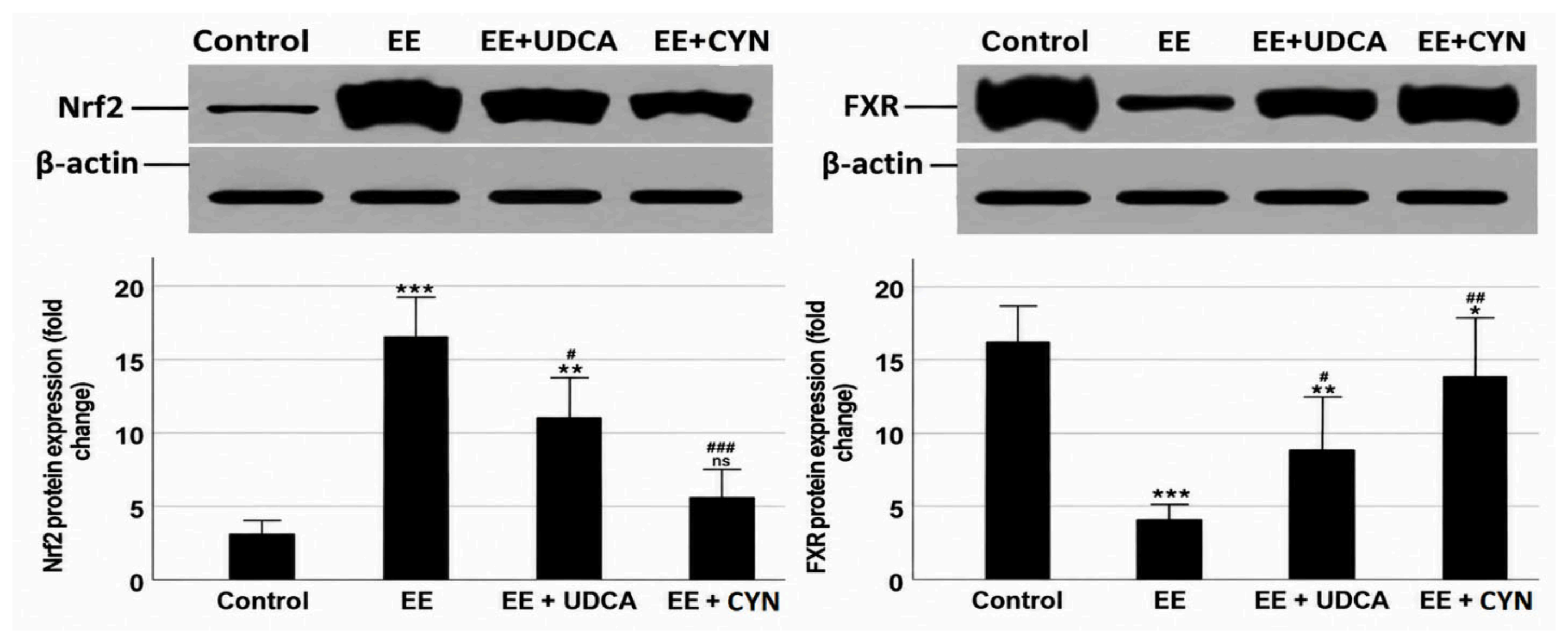
Representative Western blot bands and corresponding densitometric analysis of hepatic Nrf2 and FXR protein levels across the experimental groups, including the control, 17α-ethinylestradiol (EE), EE + ursodeoxycholic acid (UDCA), and EE + cynaroside (CYN) groups. β-actin served as the loading control. Densitometric values are expressed as fold changes relative to the control group and are presented as mean ± SD. Statistical significance was defined as * *p* < 0.05, ** *p* < 0.01, and *** *p* < 0.001 versus the control group, and ^#^ *p* < 0.05, ^##^ *p* < 0.01, and ^###^ *p* < 0.001 versus the EE group. ns, non-significant.

As shown in Figure 4, Western blot analysis demonstrated a significant increase in hepatic Nrf2 protein expression in rats treated with 17α-ethinylestradiol (EE) compared with the control group (** *p* < 0.001). Administration of ursodeoxycholic acid (UDCA) significantly reduced Nrf2 protein expression relative to the EE group (^##^ *p* < 0.01); however, expression levels remained significantly higher than those observed in the control group (*p* < 0.05). In contrast, treatment with cynaroside (CYN) markedly decreased Nrf2 protein expression compared with the EE group (^###^ *p* < 0.001), restoring its expression to a level that was not significantly different from the control group (ns).

In contrast, hepatic FXR protein expression was markedly suppressed following EE administration compared with the control group (** *p* < 0.001). Both CYN and UDCA significantly increased FXR protein expression relative to the EE-treated group (^#^ *p* < 0.05 and ^##^ *p* < 0.01, respectively). FXR expression in the CYN-treated group was partially restored and remained significantly lower than that of the control group (* *p* < 0.01), while UDCA treatment also improved FXR expression but did not restore it to control values.

### 2.7. Effects of Cynaroside on Hepatic Gene Expression

**Figure 5:**
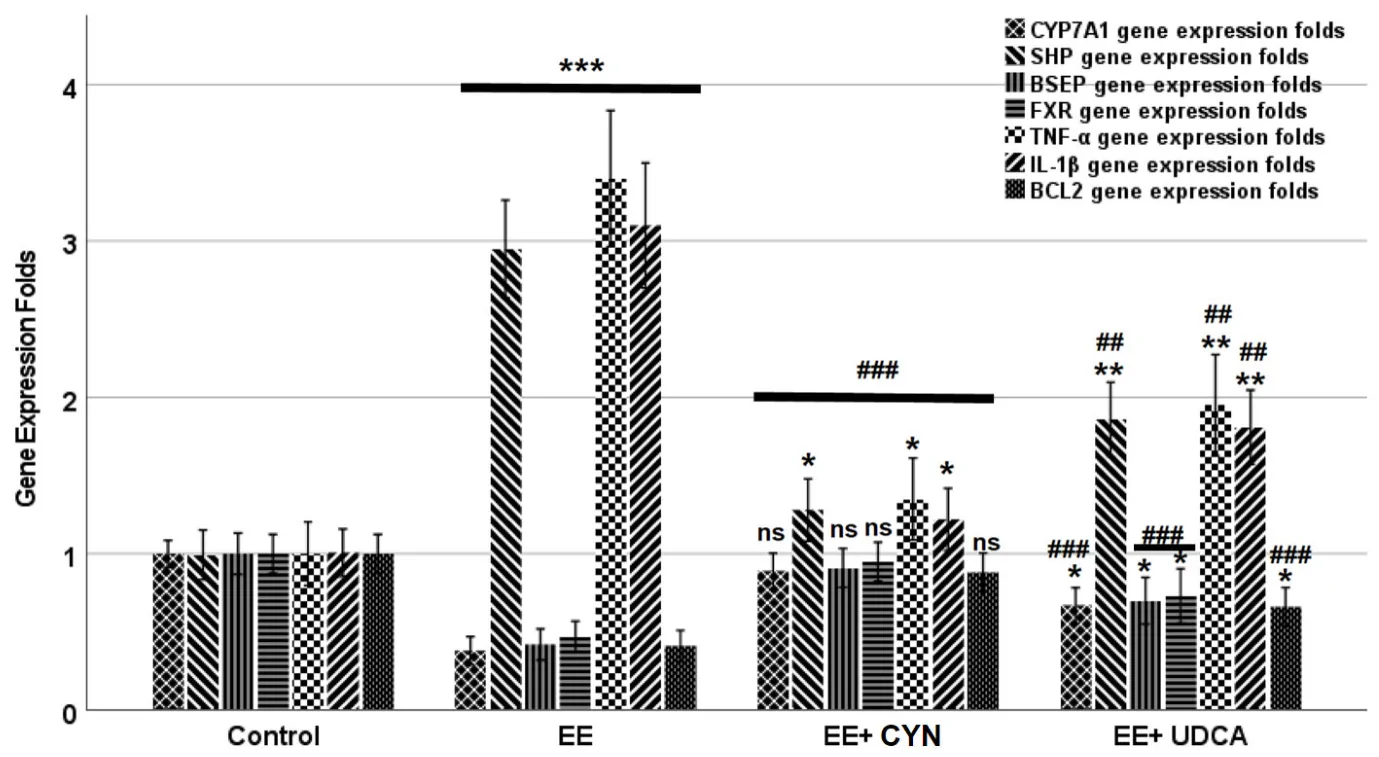
Effects of cynaroside (CYN) and ursodeoxycholic acid (UDCA) on hepatic gene transcription in the 17α-ethinylestradiol (EE)-induced cholestasis model. Relative mRNA levels of CYP7A1, SHP, BSEP, FXR, TNF-α, IL-1β, and BCL2 were determined in the control, EE, EE + CYN, and EE + UDCA groups. Data are expressed as mean ± SD (*n* = 7). Statistical significance is denoted by * *p* < 0.05, ** *p* < 0.01, and *** *p* < 0.001 versus the control group, ^##^ *p* < 0.01, and ^###^ *p* < 0.001 versus the EE group. ns, non-significant.

As illustrated in Figure 5, exposure to 17α-ethinylestradiol (EE) was associated with significant changes in the hepatic transcriptional profile of genes related to bile acid homeostasis, inflammatory responses, and cell survival. Relative to the control group, EE significantly reduced the mRNA levels of FXR, BSEP, CYP7A1, and BCL2 (*p* < 0.001), whereas SHP, TNF-α, and IL-1β transcripts were significantly elevated (*p* < 0.001).

Cynaroside (CYN) treatment was associated with an improvement in these EE-related transcriptional alterations. Compared with the EE group, CYN significantly increased the mRNA levels of FXR, BSEP, CYP7A1, and BCL2 (*p* < 0.05, *p* < 0.01, or *p* < 0.001), while reducing SHP, TNF-α, and IL-1β transcript levels. For several of the evaluated genes, no significant difference was detected between the CYN-treated and control groups (ns), suggesting partial normalization of the EE-associated transcriptional changes.

Similarly, treatment with ursodeoxycholic acid (UDCA) significantly improved the expression of all evaluated genes relative to the EE group (*p* < 0.05 or *p* < 0.01). However, most transcript levels remained significantly different from those of the control group, indicating incomplete recovery under the present experimental conditions.

### 2.8. Histopathological Evaluation of Liver Tissue

**Figure 6:**
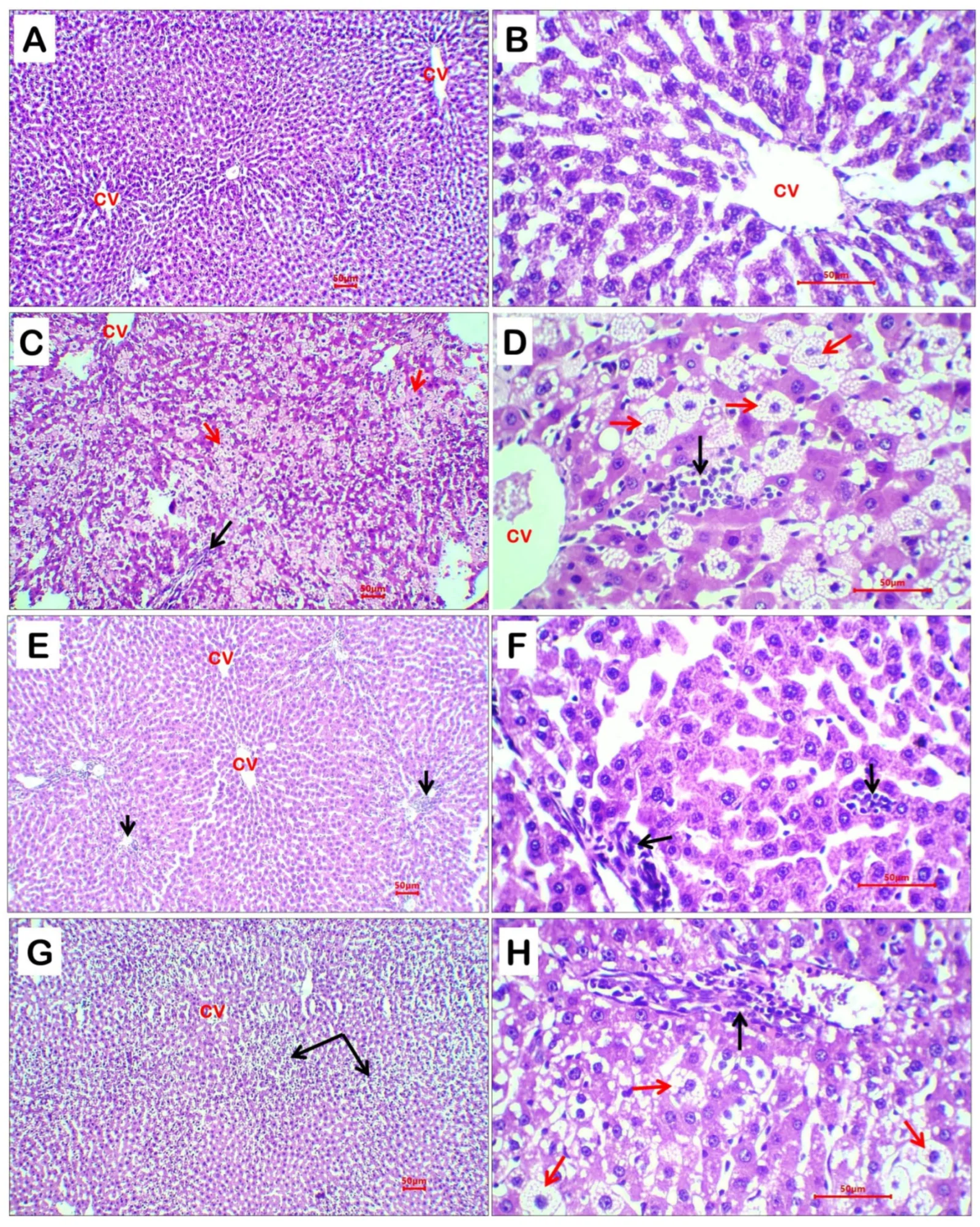
Representative H&E-stained liver sections showing the histopathological effects of cynaroside on 17α-ethinylestradiol-induced cholestatic liver injury. (**A**,**B**) Control group showing preserved hepatic architecture and normally arranged hepatocytes. (**C**,**D**) EE group showing marked hepatocellular degeneration, cytoplasmic vacuolation, inflammatory cell infiltration, and architectural disruption. Red arrows indicate hepatocellular vacuolar degeneration, whereas black arrows indicate inflammatory cell infiltration. (**E**,**F**) EE + CYN group showing substantial preservation of hepatic architecture with markedly reduced degenerative and inflammatory changes. (**G**,**H**) EE + UDCA group showing improved hepatic architecture with fewer pathological alterations than the EE group. (**A**,**C**,**E**,**G**) represent lower-magnification fields, whereas (**B**,**D**,**F**,**H**) represent higher-magnification fields. CV, central vein. Scale bars = 50 μm.

Representative hematoxylin and eosin (H&E)-stained liver sections are presented in Figure 6. Liver sections from the control group (Figure 6A,B) exhibited normal hepatic architecture with well-preserved hepatic lobules and cords of hepatocytes displaying normal cellular morphology.

In contrast, liver tissues from the 17α-ethinylestradiol (EE)-treated group (Figure 6C,D) showed marked histopathological alterations, including extensive cloudy swelling and hydropic degeneration of hepatocytes. Multifocal inflammatory cell infiltration was evident within both the portal areas and hepatic lobules, predominantly consisting of lymphocytic aggregates. No histological evidence of hepatic necrosis was observed.

Administration of cynaroside (CYN) markedly improved the histological appearance of the liver (Figure 6E,F). Most hepatic lobules retained their normal architecture, with only mild focal inflammatory cell infiltration and occasional scattered degenerative hepatocytes.

Similarly, treatment with ursodeoxycholic acid (UDCA) (Figure 6G, H) alleviated the EE-induced histopathological alterations. However, residual hepatocellular degeneration and inflammatory cell infiltration remained more evident than those observed in the CYN-treated group.

Overall, both CYN and UDCA ameliorated EE-induced hepatic histopathological injury, whereas liver sections from the CYN-treated animals exhibited histological features that more closely resembled those of the control group.

### 2.9. Immunohistochemical Expression of NF-κB p65

**Figure 7:**
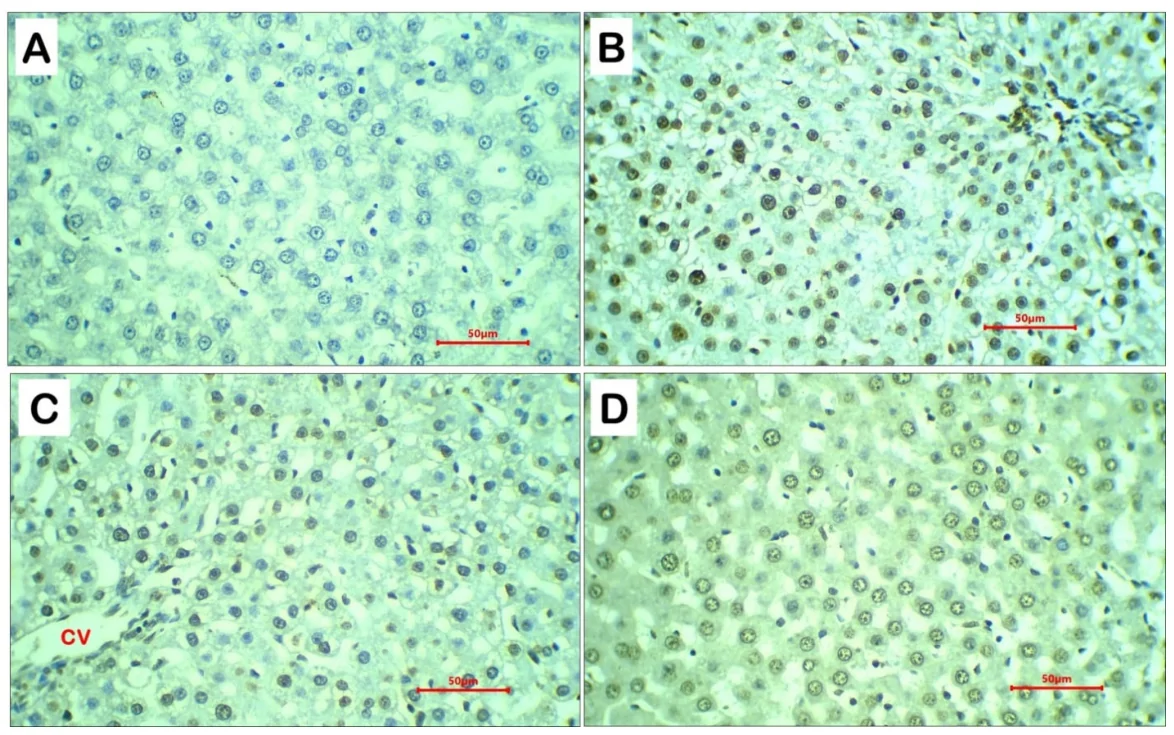
Representative immunohistochemical images illustrating NF-κB expression in hepatic sections obtained from the experimental groups. (**A**) Control group exhibiting weak or minimal NF-κB immunostaining. (**B**) EE group showing increased NF-κB-positive immunoreactivity. (**C**) E group demonstrating more pronounced NF-κB-positive staining. (**C**) EE + CYN group showing lower NF-κB immunoreactivity than that observed in the EE group. (**D**) EE + UDCA group showing reduced NF-κB immunoreactivity. Representative images were selected from comparable hepatic regions and acquired at the same magnification to facilitate comparison among groups. Brown nuclear staining indicates NF-κB-positive immunoreactivity. Scale bars = 50 μm.

Immunohistochemical staining demonstrated that NF-κB p65 expression was localized predominantly within the nuclei of hepatocytes, as indicated by brown nuclear immunoreactivity (Figure 7). Liver sections from the control group (Figure 7A) showed no detectable nuclear NF-κB p65 staining.

In contrast, liver tissues from rats treated with 17α-ethinylestradiol (EE) (Figure 7B) exhibited moderate nuclear NF-κB p65 immunoreactivity in the majority of hepatocytes, indicating marked activation of the inflammatory signaling pathway.

Treatment with cynaroside (CYN) (Figure 7C) markedly reduced NF-κB p65 immunostaining, with only weak focal nuclear positivity observed. Similarly, ursodeoxycholic acid (UDCA) treatment (Figure 7D) decreased NF-κB p65 expression compared with the EE group; however, weak-to-moderate nuclear staining remained evident.

### 2.10. Molecular Docking Analysis Within the FXR Ligand-Binding Domain

**Figure 8:**
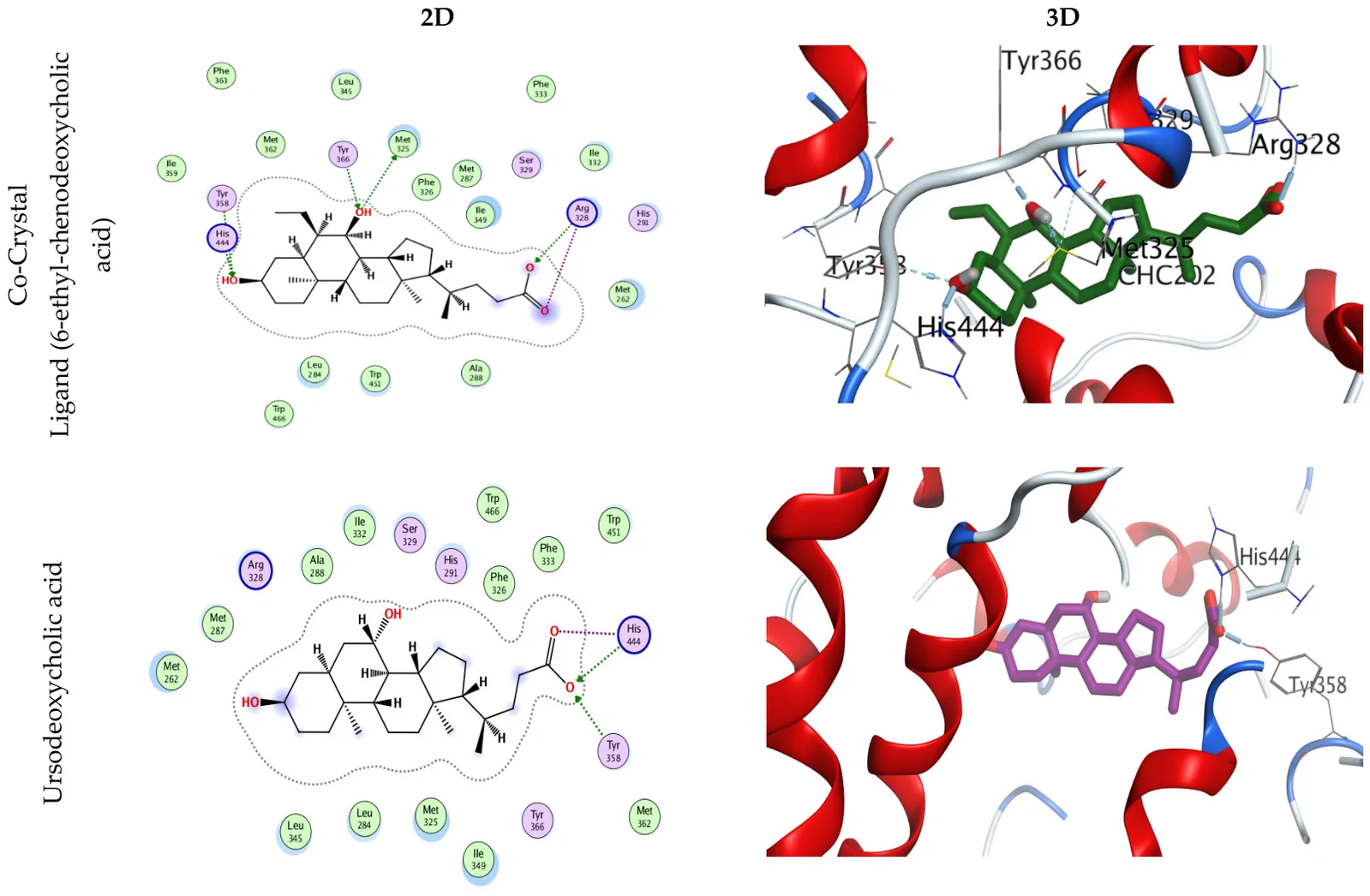

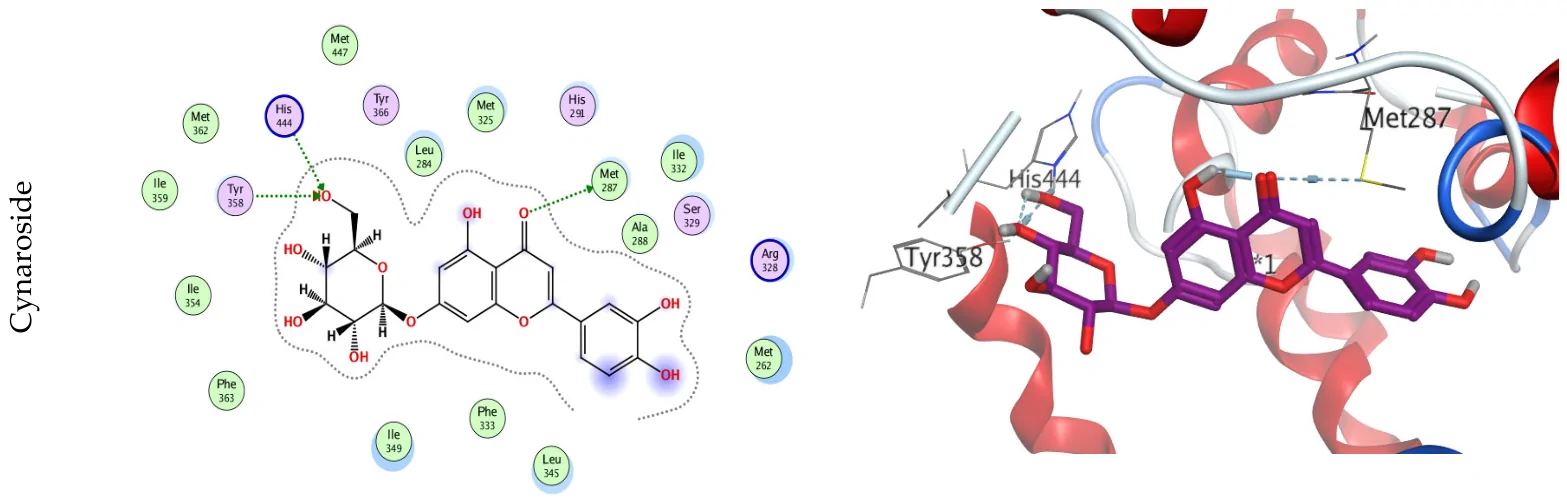
Two- and three-dimensional molecular docking interaction profiles of ursodeoxycholic acid (UDCA) and cynaroside within the ligand-binding domain of the farnesoid X receptor (FXR; PDB ID: 1OSV). The docking models illustrate the predicted binding orientations and hydrogen-bond and ionic interactions of UDCA and cynaroside with key residues within the FXR ligand-binding pocket.

An in silico docking approach was employed to characterize the predicted interaction of cynaroside (CYN) with the ligand-binding domain of human farnesoid X receptor (FXR; PDB ID: 1OSV), using ursodeoxycholic acid (UDCA) as a reference ligand. The predicted binding poses and interaction profiles of both ligands are presented in Figure 8, while the corresponding binding energies and receptor–ligand interactions are summarized in Table 3.

Validation of the docking procedure involved removing the native ligand and subsequently reproducing its binding pose within the FXR ligand-binding cavity. The predicted pose showed good agreement with the crystallographic orientation, with an RMSD value below 2.0 Å, supporting the reliability of the applied docking procedure.

Cynaroside was predicted to exhibit a favorable binding mode within the FXR ligand-binding pocket, with a predicted binding energy of −8.18 kcal/mol and an RMSD of 1.50 Å. As shown in Figure 8 and Table 3, CYN established hydrogen-bond interactions with residues located within the binding cavity, including MET287 through a hydrogen-donor interaction at a distance of 3.70 Å, TYR358 through a hydrogen-acceptor interaction at 3.14 Å, and HIS444 through a hydrogen-acceptor interaction at 2.95 Å. These interactions may contribute to the favorable accommodation of CYN within the FXR ligand-binding pocket.

For comparison, it showed a predicted binding energy of −7.95 kcal/mol with an RMSD of 1.76 Å. UDCA interacted with TYR358 through a hydrogen-acceptor interaction at 2.79 Å and with HIS444 through both hydrogen-bond and ionic interactions, with interaction distances of 2.88 and 3.12 Å, respectively. Thus, CYN showed a slightly more favorable predicted binding energy than UDCA (−8.18 versus −7.95 kcal/mol), together with a comparably low RMSD, indicating a favorable docking pose within the FXR ligand-binding domain. These computational findings provide supportive structural evidence for a potential interaction with FXR but cannot establish direct binding or receptor activation.

## 3. Discussion

The present findings indicate that cynaroside (CYN) attenuated 17α-ethinylestradiol (EE)-induced cholestatic hepatic injury, as demonstrated by improvements in serum biochemical parameters, oxidative and inflammatory markers, bile acid regulatory proteins and genes, and liver histopathology. These findings support a multifaceted hepatoprotective effect of CYN involving modulation of bile acid homeostasis and attenuation of oxidative and inflammatory disturbances. However, the molecular findings represent associations with hepatoprotection, and direct activation of specific signaling pathways was not established.

EE administration produced marked hepatomegaly and significant increases in serum AST, ALT, ALP, GGT, bilirubin, total bile acids, and cholesterol, accompanied by reductions in total protein and albumin. These changes, together with the histopathological alterations, confirmed successful induction of cholestatic liver injury and were consistent with previous reports [20]. The increases in AST and ALT indicate hepatocellular injury, whereas elevations in ALP, GGT, and bilirubin are consistent with impaired bile flow and biliary excretion. Reduced albumin and total protein may additionally reflect impaired hepatic synthetic function [28,29].

CYN substantially attenuated these biochemical abnormalities, although complete normalization was not achieved for all parameters. This effect is consistent with the reported antioxidant and anti-inflammatory properties of cynaroside, including attenuation of oxidative stress and lipid peroxidation [30,31]. The oral bioavailability of CYN is also relevant because absorption, metabolism, and systemic exposure may influence its biological effects. CYN is the 7-O-glucoside derivative of luteolin and may undergo metabolic transformation after oral administration. Although the present study demonstrated hepatoprotection following oral CYN treatment, pharmacokinetic and tissue-distribution parameters were not assessed. Therefore, the contribution of CYN and its metabolites to the observed effects remains to be clarified [32,33]. Similarly, luteolin has previously shown protective effects in experimental cholestatic liver injury [34].

Oxidative stress was evident in EE-treated animals, as shown by increased hepatic MDA and decreased GSH. Nrf2 protein expression was also increased, which may represent a compensatory response to oxidative stress; however, the concomitant oxidative damage suggests that this response was insufficient [35,36,37,38]. CYN reduced MDA and restored GSH, accompanied by lower Nrf2 expression. Because nuclear translocation and downstream targets such as HO-1 and NQO1 were not assessed, these findings do not establish direct activation or inhibition of the Nrf2 pathway. The reduction in Nrf2 may instead reflect reduced oxidative burden and diminished need for compensatory responses.

CYN also reduced NF-κB p65 expression and inflammatory mediators, including TNF-α and IL-1β, as well as hepatic MPO. These findings are consistent with reported anti-inflammatory effects of cynaroside and its ability to modulate NF-κB-associated signaling [39,40]. Because oxidative stress and inflammation are closely interconnected during hepatic injury, their concurrent improvement may contribute to the hepatoprotective effect of CYN. Nevertheless, the present data do not establish whether modulation of Nrf2 or NF-κB is a primary mechanism or a secondary consequence of reduced hepatic injury.

Disruption of bile acid homeostasis is a central feature of cholestatic liver injury and is regulated in part by FXR. In the present study, EE reduced hepatic FXR and BSEP expression and altered CYP7A1 and SHP expression, consistent with impaired bile acid regulatory processes reported in estrogen-induced cholestasis [41,42]. The reduction in FXR and BSEP was accompanied by increased serum total bile acids, supporting impaired bile acid export. CYN restored FXR and BSEP expression and reduced serum total bile acids, suggesting improvement in hepatobiliary bile acid transport and homeostasis [10,43].

The EE-associated increase in SHP despite reduced FXR, together with decreased CYP7A1, did not fully conform to the canonical FXR–SHP–CYP7A1 relationship. This may indicate involvement of compensatory or FXR-independent regulatory mechanisms, including other nuclear receptors and inflammatory pathways. CYN normalized CYP7A1 and SHP expression; however, these changes should be interpreted as modulation of selected bile acid regulatory markers rather than definitive restoration of a canonical FXR pathway [44]. Crosstalk between FXR, Nrf2, and inflammatory signaling has been reported [42,45]. The simultaneous improvement of these parameters following CYN treatment is therefore mechanistically plausible, but direct FXR transcriptional activity was not measured.

The reduction in BCL2 following EE exposure, followed by its restoration with CYN, suggests an association with improved hepatocyte survival under cholestatic conditions. Similar cytoprotective effects have been reported for flavonoids and flavone glycosides [46]. However, because other apoptotic markers were not evaluated, the present findings do not establish direct inhibition of apoptosis.

Molecular docking further provided hypothesis-generating evidence for a potential interaction between CYN and FXR. CYN showed a predicted binding energy of −8.18 kcal/mol within the human FXR ligand-binding domain (PDB ID: 1OSV), with interactions involving TYR358 and HIS444 [47,48]. These findings are consistent with the observed restoration of FXR-associated markers but do not establish direct receptor binding, modulation of FXR-related signaling, or agonistic activity. Experimental binding and functional assays are therefore required to validate this proposed interaction.

Histopathological examination provided complementary evidence of hepatoprotection. EE caused marked hepatic architectural alterations, whereas CYN treatment substantially preserved hepatic structure, with reduced inflammatory infiltration and hepatocellular degeneration. These findings were consistent with the improvements observed in biochemical, oxidative, inflammatory, and bile acid-related parameters.

UDCA, included as a reference treatment, also improved several biochemical, oxidative, inflammatory, and molecular parameters and was associated with partial histological recovery. However, because the study was not designed as a formally powered head-to-head comparison, differences between CYN and UDCA should be interpreted descriptively rather than as evidence of superiority.

Overall, CYN attenuated EE-induced cholestatic liver injury and was associated with improved liver function, reduced oxidative and inflammatory disturbances, restoration of FXR/BSEP expression, normalization of selected CYP7A1 and SHP responses, restoration of BCL2, and preservation of hepatic architecture. The docking results additionally provided structural support for a potential CYN–FXR interaction. Collectively, these findings are consistent with coordinated modulation of bile acid homeostasis, oxidative stress, inflammation, and cell-survival pathways. Nevertheless, the causal relationships among these pathways and direct receptor activation remain to be established.

Several limitations should be considered. Only one CYN dose (50 mg/kg/day) was tested, and no CYN-only group was included. In addition, the study did not assess several important bile acid regulators and transporters, including MRP2, NTCP, and FGF15/19, or perform comprehensive bile acid metabolomic profiling. Functional changes in Nrf2 expression were not established because nuclear translocation and downstream targets were not assessed. Similarly, docking provides computational rather than experimental evidence of CYN–FXR interaction. Finally, pharmacokinetic and tissue-distribution studies were not performed. Future investigations incorporating multiple CYN doses, a CYN-only group, broader bile acid profiling, pharmacokinetic analysis, and functional validation of FXR and Nrf2 signaling are warranted.

## 4. Materials and Methods

### 4.1. Materials and Drugs

Sigma-Aldrich (Merck, St. Louis, MO, USA) provided 17α-ethinylestradiol (EE; CAS No. 57-63-6) and cynaroside (luteolin-7-O-β-D-glucoside; Cat. No. 74284; purity ≥98%, HPLC). Throughout the investigation, all supplementary chemicals, solvents, and analytical reagents were of analytical quality and were utilized exactly as given by the manufacturers, requiring no extra purification.

### 4.2. Reagents and Kits

In accordance with the manufacturers’ instructions, commercially available colorimetric assay kits or enzyme-linked immunosorbent assay (ELISA) kits were used to measure serum biochemical indices, liver function markers, oxidative stress biomarkers, and inflammatory mediators. Alkaline phosphatase (ALP), aspartate aminotransferase (AST), and alanine aminotransferase (ALT) are examples of liver function markers; albumin, total protein, and direct and total bilirubin were analyzed using diagnostic kits supplied by Bio-diagnostic (Cairo, Egypt). Serum bilirubin concentrations were additionally quantified using a Bilirubin Assay Kit (Cat. No. DIBR-180; BioAssay Systems, Hayward, CA, USA), whereas total cholesterol was measured using a colorimetric assay kit (BioChain, Newark, NJ, USA).

Inflammatory status was evaluated by measuring serum tumor necrosis factor-α (TNF-α; Cat. No. RTA00-1; R&D Systems, Shanghai, China) together with hepatic myeloperoxidase (MPO; Cat. No. ab285308; Abcam, Cambridge, UK) using ELISA. Gamma-glutamyl transferase (GGT; Cat. No. MBS9343646) activity and total bile acid (TBA; Cat. No. MBS2604091) concentrations were determined using ELISA kits obtained from MyBioSource (San Diego, CA, USA).

Oxidative stress markers in the liver were quantified using liver homogenates. Malondialdehyde (MDA; Cat. No. A003-1) and reduced glutathione (GSH; Cat. No. A006-2-1) were measured with colorimetric assay kits obtained from Nanjing Jiancheng Bio-Technology Co., Ltd. (Nanjing, China). The measurements were carried out following the protocols provided by the manufacturer, and the resulting values were included in the subsequent statistical analyses.

### 4.3. Experimental Animals

Twenty-eight healthy adult female Wistar rats, aged between 8 and 10 weeks and weighing 180 to 200 g, were incorporated into this research. All experimental methods were evaluated and approved by the Sohag Institutional Animal Care and Use Committee (Sohag-IACUC), Faculty of Science, Sohag University (Approval No. SU-FS-23-26), and were executed in compliance with institutional ethical standards and the ARRIVE guidelines for documenting animal research.

Based on prior research, the sample size was established. Using comparable experimental models of estrogen-induced cholestasis, six to eight animals per group provided sufficient statistical power to detect significant biochemical and histopathological alterations [43,49].

Animals were maintained under standardized laboratory conditions at a temperature of 25 ± 2 °C and a relative humidity of 65 ± 10%, with 11–13 air exchanges per hour and a controlled 12 h light/12 h dark photoperiod. Before treatment, the rats were allowed a one-week acclimatization period. Standard laboratory chow and drinking water were provided ad libitum throughout the experiment.

### 4.4. Design of Experiments and Protocol for Treatment

Following acclimatization, female Wistar rats were randomly assigned to four experimental groups (*n* = 7 per group):

Group I (Control): Animals received the corresponding vehicles throughout the study, including subcutaneous dimethyl sulfoxide (DMSO) and oral distilled water.

Group II (EE): Rats were administered 17α-ethinylestradiol (EE; 5 mg/kg/day, subcutaneously) for 18 consecutive days to establish experimental cholestatic liver injury.

Group III (EE + CYN): Rats received EE (5 mg/kg/day, s.c.) for 18 days. Cynaroside (50 mg/kg/day, orally) was administered once daily from day 5 to day 18.

Group IV (EE + UDCA): Rats received EE (5 mg/kg/day, s.c.) for 18 days together with ursodeoxycholic acid (UDCA; 40 mg/kg/day, orally), administered once daily from day 5 to day 18.

The EE-induced cholestasis model was established according to previously validated protocols demonstrating reproducible impairment of bile acid transport and hepatobiliary function [12,50]. The cynaroside dosage was chosen because prior research in animal models of liver injury showed notable hepatoprotective, antioxidant, and anti-inflammatory properties without any indication of toxicity [23]. UDCA was included as the reference treatment because of its well-established clinical efficacy in cholestatic liver diseases through modulation of bile acid homeostasis and hepatocellular protection [51,52].

Female rats were used because this model reproduces estrogen-associated cholestatic liver injury and closely reflects the pathophysiological characteristics of estrogen-induced cholestatic disorders in humans [53,54]. Animals were monitored every day for any indications of treatment-related toxicity, behavioral changes, and clinical status throughout the trial.

After four days of EE exposure, sufficient time was allowed for the cholestatic insult to develop before treatment initiation. This experimental design was intended to model a therapeutic intervention initiated after the onset of EE-induced hepatic injury, rather than a prophylactic treatment administered before or concomitantly with the induction of cholestasis.

### 4.5. Sample Collection and Biochemical, Molecular, and Histopathological Processing

Rats were sedated and fasted for the entire night following the treatment procedure before samples were taken. After a clot formed, blood was drawn via heart puncture, and serum was separated by centrifugation at 3000 rpm for 15 min. Serum aliquots were kept at −20 °C until biochemical analysis.

Serum biochemical and inflammatory parameters were determined, including alanine aminotransferase (ALT), aspartate aminotransferase (AST), alkaline phosphatase (ALP), gamma-glutamyl transferase (GGT), total protein, albumin, total bilirubin, direct bilirubin, total bile acids (TBA), total cholesterol, and tumor necrosis factor-α (TNF-α).

The liver was removed right away after the blood was collected, cleaned with ice-cold physiological saline, gently blotted dry, and weighed. After that, the liver tissue was split into three sections. In order to measure oxidative stress and inflammatory biomarkers, such as myeloperoxidase (MPO), reduced glutathione (GSH), and malondialdehyde (MDA), the first part was homogenized. For Western blot investigation of FXR and Nrf2 protein expression, the second portion was snap-frozen and kept at −80 °C. For histological analysis and immunohistochemical evaluation of NF-κB expression, the remainder was preserved in 10% neutral-buffered formalin.

### 4.6. Western Blot Analysis of NRF2 and FXR Protein Expression

Using a SCILOGEX D160 homogenizer (SCILOGEX, Rocky Hill, CT, USA; 0.1–50 mL sample capacity) at 4 °C, frozen liver tissue samples (50–100 mg) were weighed and homogenized in 1 mL of ice-cold RIPA lysis buffer (Thermo Fisher Scientific, Waltham, MA, USA; Cat. No. 89900) supplemented with protease and phosphatase inhibitor cocktails. Total protein lysates were obtained by centrifuging the homogenates at 12,000× *g* for 15 min at 4 °C. The Pierce™ BCA Protein Assay Kit (Thermo Fisher Scientific, Waltham, USA; Cat. No. 23225) was used to measure total protein concentrations in accordance with the manufacturer’s instructions. Using a semi-dry transfer system, equal amounts of protein (30 μg) were separated by 10% SDS-polyacrylamide gel electrophoresis and electrotransferred onto PVDF membranes (Model 8050, Millipore, Bedford, MA, USA).

The membranes were blocked for 1 h at room temperature with 5% non-fat milk prepared in TBS-T, followed by overnight incubation at 4 °C with the specified primary antibodies, including NRF2 (rabbit monoclonal, 1:1000; Abcam, Cambridge, UK, Cat. No. ab62352), FXR (rabbit monoclonal, 1:1000; Cell Signaling Technology, Cat. No. #12295), and β-actin (mouse monoclonal, 1:5000; Sigma-Aldrich, Cat. No. A5316). β-actin was used as the loading control for normalization.

Following the washing steps, the membranes were exposed to the corresponding HRP-labeled secondary antibody (1:5000; Abcam, Cambridge, UK, Cat. No. ab6721) for 1 h at room temperature. Protein bands were subsequently visualized using an enhanced chemiluminescence substrate (ECL; Thermo Fisher Scientific, Waltham, USA; Cat. No. 32209) and imaged with a ChemiDoc MP Imaging System (Bio-Rad) using Image Lab software (Bio-Rad; version 4.1). Densitometric analysis was performed using ImageJ (version 1.54), and NRF2 and FXR signals were normalized to β-actin and reported as fold changes relative to the control group.

### 4.7. RNA Isolation and Quantitative Real-Time PCR (qRT-PCR)

Total RNA was isolated from frozen liver tissue using TRIzol™ Reagent (Invitrogen, Fisher Scientific, Waltham, USA; Cat. No. 15596026) in accordance with the manufacturer’s instructions. RNA yield and purity were assessed spectrophotometrically using a NanoDrop™ instrument (Thermo Fisher Scientific, Waltham, USA), and samples exhibiting A260/A280 ratios of 1.8–2.0 were considered suitable for downstream analysis. For cDNA synthesis, 1 μg of total RNA was reverse-transcribed using the RevertAid First Strand cDNA Synthesis Kit (Thermo Scientific, Vilnius, Lithuania; Cat. No. K162). Quantitative real-time PCR was performed using PowerUp™ SYBR™ Green Master Mix (Applied Biosystems, Foster City, CA, USA; Cat. No. A25742) on an Applied Biosystems StepOnePlus™ Real-Time PCR System. Transcript levels of CYP7A1, SHP, BSEP, FXR, TNF-α, IL-1β, and BCL2 were determined in triplicate. Relative mRNA expression was calculated using the 2^−ΔΔCt^ comparative method, with β-actin serving as the endogenous reference gene. Gene-specific primers were synthesized by Macrogen Inc. (Seoul, Republic of Korea), and the corresponding nucleotide sequences are provided in Table 4.

### 4.8. Histological Examination Using Hematoxylin and Eosin (H&E) Staining

Liver samples were preserved using 10% neutral-buffered formalin and processed routinely for paraffin embedding. Following tissue dehydration and xylene clearing, paraffin blocks were prepared and sectioned at 4–5 μm thickness with a rotary microtome. The obtained sections were placed on glass slides, deparaffinized, and rehydrated before H&E staining according to the method of Cardiff et al. (2014) [55]. The stained sections were then dehydrated, cleared, and coverslipped using a resinous mounting medium. Tissue morphology and pathological changes were examined under a bright-field light microscope, and representative microscopic fields were captured at suitable magnifications.

### 4.9. Immunohistochemical Analysis of NF-κB p65 Expression

Paraffinized liver tissue sections (4–5 μm thick) were processed for immunohistochemical detection of NF-κB p65. Sections were first treated with xylene to remove paraffin and subsequently rehydrated using a descending ethanol series. Heat-induced epitope retrieval was carried out in 10 mM citrate buffer (pH 6.0) for 20 min. Tissue sections were treated with 3% hydrogen peroxide for 10 min to quench endogenous peroxidase activity. Nonspecific binding sites were then blocked using 5% normal goat serum for 30 min at room temperature.

The sections were incubated at 4 °C overnight with rabbit anti-NF-κB p65 primary antibody (1:200; Cell Signaling Technology, CA, USA; Cat. No. 8242). Following washing to remove unbound antibody, an HRP-linked secondary antibody (1:500; Abcam, Cambridge, UK, Cat. No. ab6721) was applied for 1 h at room temperature. The immunoreaction was developed with 3,3′-diaminobenzidine (DAB), and the sections were counterstained with hematoxylin. Subsequently, the slides were dehydrated, cleared with xylene, and permanently mounted.

NF-κB p65 staining was visualized by bright-field microscopy. For quantitative assessment, five randomly selected high-power fields (HPFs) from each section were analyzed, and the percentage of nuclei showing positive immunostaining was calculated using ImageJ software (version 1.54g). Histological staining and quantitative image analysis were conducted following the approach reported by Allen and Van Waes (2015) [56].

### 4.10. In Silico Molecular Docking Study

A molecular docking approach was used to assess the predicted interaction of cynaroside with the ligand-binding domain of human farnesoid X receptor (FXR). The crystallographic structure of human FXR complexed with its co-crystallized ligand was obtained from the Protein Data Bank (PDB ID: 1OSV). The receptor structure was prepared using AutoDock Tools (v1.5.7), including removal of water molecules and non-essential heteroatoms while preserving the protein framework. Polar hydrogen atoms were subsequently added, and Kollman charges were assigned to the receptor before docking.

For validation of the docking setup, the ligand present in the original crystal structure was removed from the FXR binding cavity and subsequently docked again using the identical computational parameters. The predicted pose was then compared with the experimentally determined crystallographic conformation. The close agreement between the two orientations, reflected by an acceptable RMSD value, supported the validity and reproducibility of the applied docking protocol.

The molecular structures of cynaroside and ursodeoxycholic acid (UDCA) were obtained from the PubChem database. Prior to docking, the ligand structures were subjected to geometry optimization using the MMFF94 force field in Avogadro, followed by conversion to the PDBQT file format.

Molecular docking was subsequently carried out with AutoDock 4.2 using the Lamarckian Genetic Algorithm (LGA). The search space was defined by a 60 × 60 × 60 Å grid box with a grid spacing of 0.375 Å, positioned over the native ligand-binding cavity. For each ligand, 50 independent docking trials were performed. The resulting poses were evaluated based on binding free energy (ΔG, kcal/mol) and RMSD, and the pose showing the most favorable binding energy with an acceptable RMSD was retained for subsequent analysis.

The predicted binding characteristics of cynaroside were assessed in relation to those of the co-crystallized ligand and UDCA as the reference compound. The docked protein–ligand complexes were subsequently analyzed to characterize hydrogen-bonding, hydrophobic, electrostatic, and π-associated interactions. Molecular interaction patterns and docking poses were inspected and graphically represented using Discovery Studio Visualizer 2021 and PyMOL (v2.5).

### 4.11. Statistical Analysis

Data are expressed as mean ± standard deviation (SD). Statistical analyses were performed using IBM SPSS Statistics for Windows, version 27.0 (IBM Corp., Armonk, NY, USA). Before analysis, the data were evaluated for normality and homogeneity of variance. Group differences were analyzed using one-way analysis of variance (ANOVA), followed by Tukey’s post hoc test for multiple pairwise comparisons. Statistical significance was established at a two-tailed *p* < 0.05.

## 5. Conclusions

Our findings indicate that cynaroside mitigates liver injury induced by 17α-ethinylestradiol, as reflected by favorable changes in hepatic biochemical parameters, markers of oxidative stress and inflammation, selected regulators of bile acid homeostasis, and liver histopathology. CYN treatment was associated with restoration of hepatic FXR and BSEP expression and normalization of CYP7A1 and SHP expression, supporting modulation of hepatic bile acid regulatory processes. However, these findings do not establish direct modulation of FXR-related signaling or restoration of a canonical FXR–SHP–CYP7A1 pathway. Similarly, changes in Nrf2 protein expression should be interpreted as alterations associated with the oxidative-stress response rather than definitive evidence of functional Nrf2 pathway activation.

The present findings extend the available evidence regarding the hepatoprotective potential of luteolin-related flavonoids and identify CYN as a candidate for further investigation in experimental estrogen-induced cholestatic liver injury. Nevertheless, the relative contribution of CYN and its potential metabolites, as well as the functional relevance of the predicted CYN–FXR interaction, remains to be established. Further studies incorporating pharmacokinetic assessment, dose–response designs, appropriate functional FXR and Nrf2 assays, and broader bile acid regulatory analyses are warranted to clarify the molecular and pharmacological basis of CYN-mediated hepatoprotection.

## 6. Future Perspectives

Future studies should further characterize the molecular and pharmacological basis of CYN-mediated hepatoprotection by examining downstream components of the FXR-associated bile acid regulatory network and the Nrf2 antioxidant response. Particular attention should be given to additional bile acid transporters and regulators, including multidrug resistance-associated protein 2 (MRP2), sodium taurocholate cotransporting polypeptide (NTCP), and other pertinent pathway components, along with downstream antioxidant markers such as heme oxygenase-1 (HO-1) and NAD(P)H quinone dehydrogenase 1 (NQO1). Comprehensive bile acid profiling and metabolomic analyses would also help determine how CYN influences bile acid composition and hepatic metabolic homeostasis.

The predicted interaction between CYN and FXR should be further evaluated using direct receptor-binding and functional approaches, including FXR reporter assays and other complementary receptor-based assays. Molecular dynamics simulations could further characterize the persistence and stability of the predicted ligand–receptor complex; however, such findings would still represent computational evidence and should be considered supportive of, rather than a substitute for, experimental validation. In parallel, pharmacokinetic and tissue-distribution studies are warranted to characterize CYN and its metabolites following oral administration and to clarify hepatic exposure in relation to the administered dose.

Further investigations may also explore formulation and drug-delivery strategies aimed at improving the absorption, stability, and hepatic availability of CYN. Before any clinical translation can be considered, comprehensive dose–response studies, long-term safety and toxicity evaluations, and appropriately designed clinical studies will be necessary to establish the potential therapeutic value and safety profile of CYN in cholestatic liver disorders.

## Figures and Tables

**Table 1:** Effect of CYN and UDCA on liver function biomarkers in EE-induced hepatic injury.

Parameters/Groups	Control	EE	EE + CYN	EE + UDCA
AST (U/mL)	51.73 ±1.92	199.61 ± 3.58 ***	68.71 ± 2.41 *^,###^	85.30 ± 3.07 **^,###^
ALT (U/mL)	42.94 ± 2.77	153.49 ± 2.41 ***	62.41 ± 2.28 **^,###^	68.47 ± 2.63 **^,###^
ALP (U/L)	45.49 ± 2.21	165.36 ± 3.24 ***	55.03 ± 2.45 *^,###^	65.40 ± 3.36 **^,###^
GGT (U/L)	28.69 ± 2.46	135.91 ± 3.89 ***	56.30 ± 2.55 ***^,###^	70.78 ± 2.99 ***^,###^
Total Protein (g/dL)	9.30 ± 0.71	6.00 ± 0.43 ***	7.71 ± 0.71 **^,##^	7.35 ± 0.30 **^,##^
Albumin (g/dL)	4.54 ± 0.50	3.19 ± 0.22 ***	4.44 ± 0.60 ^ns,###^	3.62 ± 0.25 **^,ns^
Direct Bilirubin (mg/dL)	0.13 ± 0.03	1.20 ± 0.06 ***	0.24 ± 0.03 *^,###^	0.73 ± 0.12 **^,##^
Total Bilirubin (mg/dL)	0.49 ± 0.03	2.57 ± 0.29 ***	0.83 ± 0.05 *^,###^	1.25 ± 0.05 **^,##^

Data are expressed as mean ± standard deviation (SD) (*n* = 7). For different experimental groups: control, ethinylestradiol (EE), EE treated with cynaroside (CYN), and EE treated with ursodeoxycholic acid (UDCA). AST: aspartate aminotransferase; ALT: alanine aminotransferase; ALP: alkaline phosphatase; GGT: gamma-glutamyl transferase; CYN: Cynaroside; UDCA: ursodeoxycholic acid; EE: ethinylestradiol. Statistical comparisons were performed using one-way ANOVA followed by Tukey’s post hoc test. * Significant vs. control group (* *p* < 0.05); ** *p* < 0.01; *** *p* < 0.001. ^#^ Significant vs. EE group ^##^ *p* < 0.01; ^###^ *p* < 0.001. ns: non-significant vs. control group.

**Table 2:** Effect of CYN and UDCA on oxidative stress markers in EE-induced hepatic injury.

Parameters/Groups	Control	EE	EE + CYN	EE + UDCA
MDA (nmol/mg tissue)	0.37 ± 0.03	0.67 ± 0.02 ***	0.43 ± 0.04 ^ns,###^	0.54 ± 0.06 **^,##^
GSH (mg/mg protein)	1.11 ± 0.05	0.62 ± 0.03 ***	0.92 ± 0.03 *^,###^	0.71 ± 0.03 **^,#^

Data are expressed as mean ± SD (*n* = 7) for control, ethinylestradiol (EE), EE treated with cynaroside (CYN), and EE treated with ursodeoxycholic acid (UDCA) groups. MDA: malondialdehyde; GSH: reduced glutathione; CYN: Cynaroside; UDCA: ursodeoxycholic acid; EE: ethinylestradiol. Statistical comparisons were performed using one-way ANOVA followed by Tukey’s post hoc test. * Significant vs. control group (* *p* < 0.05); ** *p* < 0.01; *** *p* < 0.001. ^#^ Significant vs. EE group (^#^ *p* < 0.05); ^##^ *p* < 0.01; ^###^ *p* < 0.001. ns: non-significant vs. control group.

**Table 3:** Binding energies and molecular interaction profiles of ursodeoxycholic acid and selected cynaroside phytoconstituents docked into the active site of the farnesoid X receptor (FXR) (PDB ID: 1OSV).

	Ligand	Receptor	Interaction	Distance	E (kcal/mol)	S (kcal/mol)	RMSD
Ursodeoxycholic acid	O 66	TYR 358	H-acceptor	2.79	−3.5	−7.95	1.76
	O 66	HIS 444	H-acceptor	2.88	13.8		
	O 66	HIS 444	Ionic	2.88	−5.3		
	O 67	HIS 444	Ionic	3.12	−3.7		
Cynaroside	O 14	MET 287	H-donor	3.70	−0.9	−8.18	1.5
	O 39	TYR 358	H-acceptor	3.14	−0.5		
	O 39	HIS 444	H-acceptor	2.95	−3.0		

**Table 4:** Oligonucleotide primer sequences used for quantitative real-time PCR.

Gene	Forward Primer (5′–3′)	Reverse Primer (5′–3′)
CYP7A1	GTGATGTTTGAAGCCGGATATC	TTTATGTGCGGTCTTGAACAAG
SHP	GTCCGACTATTCTGTATGCACT	CTACTGTCTTGGCTAGGACATC
BSEP	ATGAAGCCATTGCCGACCAGATG	GACTGACAGCGAGAATCACCAAGG
FXR	GCTAATGAGGACGACAGCGAAGG	GTCTGTTGGTCTGCCGTGAGTTC
TNF-α	GCGACGTGGAACTGGCAGAAG	GCCACAAGCAGGAATGAGAAGAGG
IL-1β	GCCCATCCTCTGTGACTCAT	AGGCCACAGGTATTTTGTCG
BCL2	GGGATGCCTTTGTGGAACTA	AGCCTGCAGCTTTGTTTCAT
β-actin	GGCATGGACTGTGGTCATGAG	TGCACCACCAACTGCTTAGC

## Data Availability

The data supporting the findings of this study are provided within the published article.

## Abbreviations

ALP	Alkaline Phosphatase
ALT	Alanine Aminotransferase
AST	Aspartate Aminotransferase
CYN	Cynaroside
EE	Ethinylestradiol
FXR	Farnesoid X Receptor
GGT	Gamma-Glutamyl Transferase
GSH	Reduced Glutathione
MDA	Malondialdehyde
MPO	Myeloperoxidase
NF-κB	Nuclear Factor Kappa B
Nrf2	Nuclear Factor Erythroid 2–Related Factor 2
TBA	Total Bile Acid
TNF-α	Tumor Necrosis Factor-alpha
UDCA	Ursodeoxycholic Acid
